# Non-coding RNA regulates phage sensitivity in *Listeria monocytogenes*

**DOI:** 10.1371/journal.pone.0260768

**Published:** 2021-12-20

**Authors:** Yuan Tian, Liting Wu, Mengya Zhu, Zhenquan Yang, García Pilar, Hongduo Bao, Yan Zhou, Ran Wang, Hui Zhang

**Affiliations:** 1 School of Food and Biological Engineering, Jiangsu University, Zhenjiang, China; 2 Jiangsu Key Laboratory of Food Quality and Safety-State Key Laboratory Cultivation Base of MOST, Jiangsu Academy of Agricultural Sciences, Nanjing, China; 3 College of Food Science and Engineering, Yangzhou University, Yangzhou, China; 4 Instituto de Productos Lácteos de Asturias (IPLA-CSIC), Asturias, Spain; University of Copenhagen, DENMARK

## Abstract

Non-coding RNAs (ncRNAs) have gained increasing attention as their diverse roles in virulence and environmental stress in *Listeria monocytogenes* have become clearer. The ncRNA *rliB* is an atypical member of the CRISPR family, conserved at the same genomic locus in all analyzed *L*. *monocytogenes* genomes and also in other *Listeria* species. In this study, *rliB* defective mutants (Lm3-22-Δ*rliB*) were constructed by homologous recombination. The growth cycle of Lm3-22-Δ*rliB* mutants was slower than that of wild-type Lm3-22. The sensitivity of Lm3-22-Δ*rliB* to the *Listeria* phage vB-LmoM-SH3-3 was significantly increased, and the efficiency of plaque formation was enhanced by 128 fold. Compared with wild type, the adhesion and invasion of Lm3-22-Δ*rliB* decreased significantly (9.3% and 1.33%, respectively). After 4 hours of infection, the proliferation of Lm3-22-Δ*rliB* in RAW264.7 cells also decreased significantly. Transcription level of invasion-related surface proteins showed that the internalin genes lmo0610 and lm0514, and the peptidoglycan binding protein gene lmo1799 in Lm3-22-Δ*rliB* were significantly increased. In addition, after interaction with phage, the transcription levels of *inlA*, lmo0610, lmo1799, lmo2085, and lmo0514 in Lm3-22-Δ*rliB* cells were significantly upregulated, while *inlB* was downregulated, compared with Lm3-22 control group with phage treatment. Therefore, *rliB* deletion effectively regulated the interaction between *Listeria* and phage, weaken its invasion ability, and provided a new theoretical basis for biocontrol of phage.

## 1. Introduction

*Listeria monocytogenes*, a Gram-positive bacterium, is responsible for almost all cases of human listeriosis and can cause severe illness in susceptible individuals with an overall 30% mortality rate [1]. A number of virulence-related genes responsible for bacterial entry and replication within host cells have been identified in *L*. *monocytogenes*. Several listerial surface proteins including LPXTG motif, GW motif, and LRR protein, play key roles in listerial interactions with mammalian host cells. Intracellular putative cell wall-associated genes *inlA*, lmo0610, lmo0514, lmo1799 and lmo2085, all of which encode products containing the LPXTG motif [2,3]. Internalin inlB (lmo0434), which encode products containing the GW motif, allows *L*. *monocytogenes* entry into nonpolarized epithelial cells in vitro [4], and it cooperates with InlA during placental invasion in vivo [5,6].The internalins A and B, encoded within a single locus in the *L*. *monocytogenes* genome, are two major surface molecules driving bacterial entry into host cells [7]. Both InlA and InlB are regulated by PrfA, a master transcriptional activator whose expression is under the control of an RNA thermosensor, by stress sigma factor SigB, or by two-component systems [8–11].

Noncoding RNAs (ncRNAs) regulating virulence have been identified in most pathogens. The identification of mRNA targets of ncRNA is essential for understanding their functions. Bacterial small ncRNAs play critical roles as regulators of gene expression in prokaryotes and eukaryotes, and respond to stressful conditions, environmental changes, and pathogenesis [12]. NcRNA Rli60 plays important roles in regulating the adaptability of *L*. *monocytogenes* to environmental stresses and biofilm formation [13]. The rli87 of *L*. *monocytogenes* displays an important regulatory role in response to temperature (30 and 42°C) and alkaline stresses [14]. Small RNA teg49 is derived from a *sarA* transcript and regulates virulence genes independent of SarA in *Staphylococcus aureus* [15]. In *L*. *monocytogenes*, the ncRNA *rliB* has five repeats of 29 nt spaced by 35–36 nt and is an atypical member of the CRISPR family that is conserved at the same genomic locus in all analyzed *L*. *monocytogenes* genomes, as well as also in other *Listeria* species. However, no cas genes have been found in the neighborhood of *rliB* [16]. NcRNAs usually regulate gene expression either by pairing to mRNAs and affecting their stability and/or translation or by binding to proteins and modifying their activity [17]. Previous studies have shown that *rliB* is stably bound by *Listeria* polynucleotide phosphorylase (PNPase), which is likely responsible for its processing into a mature form [10]. The deletion of *rliB* impairs virulence in mice [18].

Although almost all *Listeria* strains are susceptible to most of the common antibiotics, the cure rate is only ~70% [19]. Nowadays, an increased resistance of *L*. *monocytogenes* to many antimicrobial agents has been increasingly observed [20,21]. Combining safe, selective antimicrobial treatments, such as phage, with conventional packaging, is expected to dramatically reduce the rates of foodborne illness by actively inhibiting these organisms and providing protection beyond the end of production processes [22]. In this study, we found that the deletion of *rliB* not only affected the invasion ability of *L*. *monocytogenes*, but also enhanced the sensitivity against phage, meaning that ncRNAs can regulate the interaction between phage and their host. This study mainly analyzes that whether *rliB* can regulate the interaction of phage and host.

## 2. Material and methods

### 2.1. Bacteria, phage, growth conditions

*L*. *monocytogenes* Lm008 and Lm3-22 were isolated from pig hind leg meat and sewage in a slaughterhouse of Jiangsu Huai’an meat Co., Ltd, respectively. *Escherichia coli* DH5α was purchased from Stratagene (La Jolla, CA, USA) and used for transformation. The shuttle vector pKSV7 was kindly provided by Dr. Guoqiang Zhu (Yangzhou University, Yangzhou, China) and used to construct the mutant *L*. *monocytogenes* Lm3-22. *Listeria* phage vB-LmoM-SH3-3 was previously isolated from the sewage of a pig farm in Jiangsu, China [23].

### 2.2. Construction of a recombinant shuttle vector

PCR primers were designed based on the nucleotide sequence of the *rliB* (lmos21) gene (NP_464605.1). All primers used in this study were synthesized by Sangon Biotech Ltd (Shanghai, China). The *rliB* deletion sequence was composed of 283 bp of sequence downstream of *lmo0509* and 284 bp upstream of *lmo0510*, with a 360-bp deletion of *rliB* in the middle. It was synthesized and cloned into a pUC57 vector by Sangon Biotech Co. The Δ*rliB* fragment was purified and digested with the restriction enzymes *Eco*RI and *Bam*HI. The purified fragments of Δ*rliB* were then inserted into the respective sites in the plasmid pKSV7. These ligation products were then transformed into DH5α competent cells and screened using ampicillin (50 μg/mL) to generate pKSV7-Δ*rliB*.

### 2.3. Generation of *L. monocytogenes* mutant strain by allelic exchange with recombinant shuttle vector

The recombinant shuttle vector pKSV7-Δ*rliB* was used to transform the *L*. *monocytogenes* strain Lm3-22 by electroporation. Then, add 1 mL of BHI broth and incubated at 37°C for 4 h. Plate cells onto BHI agar, containing 10 μg/mL chloramphenicol (Sigma, St. Louis, MO, USA). Incubate at 30°C for 48 h. Transformants were incubated at 41°C in Brain Heart Infusion (BD Difco, USA) medium containing 10 μg/mL chloramphenicol (Sigma, St. Louis, MO, USA) to direct chromosomal integration of the plasmid DNA by homologous recombination. Chromosomal integration of the reporter gene was confirmed by PCR, using the primer pair rliB-1/rliB-2. These amplicons were further verified by sequencing and the resulting strain was named Lm3-22-Δ*rliB*. For complementation, the entire *rliB* and flanking regions were amplified using primer rliB::B-1 and rliB::B-2 (Table 1). PCR products were *Bam*HI–*Xba*I digested and ligated to the site-specific integration vector pPL2 [24]. The resultant plasmid was designated pPL-rilB. pPL-rilB was transformed in to *E*. *coli* DH5α competent cells and the resulting strain was mated into Lm3-22-Δ*rliB*::B. Primers rliB-1 and rliB-2 were used to confirm the presence of *rliB* in the complemented strain.

**Table 1:** Oligonucleotide primers sets used in this study.

Gene	Primer	Sequence (5′-3′)
*inlA*	InlA-1	TAACCAAATAAGTAACATCAGTCC
	InlA-2	GCGCCAGTCACATTTTTCAC
*inlB*	InlB-1	CTGGACTAAAGCGGAAAACC
	InlB-2	ATTGGCGCTGACATAACGAGT
*lmo0610*	lmo0610-1	GTTTAAAAGCAACGCCAACACA
	lmo0610-2	GTGGCGTCGGAGGTTCATT
*lmo1799*	lmo1799-1	GATGATGGTAGCGCCTGTCA
	lmo1799-2	CCGCCAATTCCTAAATAAGCAA
*lmo2085*	lmo-1	GTATTCAGCAAGATAGCGAAGAACCT
	lmo-2	TCGTCGTTATTCCCGCATCTA
*lmo0514*	lmo0514-1	TGCTGCAGGACTCAAAGCAA
	lmo0514-2	TGTCCACTGTCGCTTGTAGTCA
*rliB*	rliB-1	ACGAATCTTAGGCCGCGCAAT
	rliB-2	GATTGTTGCTAACAATAATAT
*rliB*::B	rliB::B-1	GCTTGGGAAATCTTCCGGAT
	rliB::B-2	ATTAGCTTACCTTCCCTTAAAT

### 2.4. RliB mutant growth curve and phage lysis effect analysis

The mutant Lm3-22-ΔrliB and wild-type Lm3-22 were inoculated on BHI agar (Cm, 10 μg/ml) and cultured overnight at 37°C. Single colonies were then transfer to BHI broth containing chloramphenicol (10 μg/mL) and cultured at 37°C with shaking at 200 rpm until they reached the logarithmic growth period, OD_600_ ≈ 0.6. The cultures were then reinoculated into BHI broth at 1:100 (V/V) and incubated at 30°C, and the OD_600_ value was measured every 30 min. Host bacteria were then infected with phage at an MOI of 100, and the concentration (OD_600_ value) of host bacteria was measured every 30 minutes to determine the lysis effect of phage on mutant Lm3-22-ΔrliB and Lm3-22, respectively.

### 2.5. Efficiency of plaquing (EOP) assays

Phage propagation was performed as described previously [25]. Phage lysate was first filtered through a 0.22 μm membrane and stored at 4°C. The phage titer was determined and an EOP assay was performed as described by Sanders and Klaenhammer [26] previously. Briefly, phage vB-LmoM-SH3-3 with different dilutions (1×10^9^ PFU/mL) were mixed with Lm008, Lm3-22-Δ*rliB* and Lm3-22, respectively. The titers of phage vB-LmoM-SH3-3 from different host were determined and refer to Lm008. The EOP was calculated by ratio of tested strain to reference strain and determining the average for three independent assays.

### 2.6. Examination of adhesion, invasion, intracellular survival, and proliferation of rliB mutant

Cell adhesion, invasion, intracellular survival, and proliferation were examined in the mouse macrophage cell line RAW264.7. RAW264.7 cells were cultured in 6-well plates in Dulbecco’s modified Eagle medium (DMEM) (HyClone) containing 10% fetal bovine serum (FBS, HyClone) at 37°C with 5% CO_2_. Half of a milliliter of Lm3-22-ΔrliB and Lm3-22 (5×10^7^ CFU/mL) were used to inoculate a 6-well plate containing 5×10^5^ cells/mL in each well for 1 hour, respectively. The mixed culture was then collected and used for counting bacterial cell numbers. Cells in each well were washed twice with PBS and resuspended in 1 mL DMEM culture medium containing gentamicin (100 μg/mL) to determine the number of adherent bacteria. After washing twice with 2 mL PBS, the cells were recultured in 2 mL DMEM culture medium containing (100 μg/mL) gentamicin for 2 to 12 hours. Cells were washed twice with PBS and lysed in 0.25% Triton X-100 (Amresco Inc., USA). Cell lysates were then diluted and cultured overnight. The number of Lm3-22-ΔrliB and Lm3-22 cells were then counted and used to calculate the adhesion rate, invasion rate, and viable intracellular bacterial number. Each experiment was repeated three times.

### 2.7. RNA extraction, cDNA synthesis, and quantitative reverse transcription real-time PCR (qRT-PCR)

To clarify the regulation effect of *rliB* between the host and phage, *L*. *monocytogenes* surface protein (LPTXG) was analyzed by qRT-PCR. The transcription levels of LPTXG protein encoding genes (i.e., *InlA*, *InlB*, lmo0610, lmo1799, lmo2085, and lmo0514) were measured using qRT-PCR (LightCycler, Roche) and reagents (SYBR® Premix Dimer Eraser™ kit; QIAGEN, Germany). In brief, 1mL Lm3-22-Δ*rliB* and Lm3-22 (10^8^ CFU/mL) cells were mixed with the phage vB-LmoM-SH3-3 (10^9^ PFU/mL). After incubating at 37°C for 120 min, the mixture was centrifuged at 10000 ×g for 5 min, with Lm3-22-Δ*rliB* and Lm3-22 without phage being used as a control group, and the pellet was collected and stored at -20°C. Total RNA was extracted from the mixture using Trizol (Vazyme Biotech) according to manufacturer’s instruction. Reverse transcription was carried out using a Reverse Transcription Kit (Vazyme Biotech) according to the manufacturer′s instructions. Quantitative real-time PCR was used to evaluate the transcription of *InlA*, *InlB*, Lmo0610, Lmo1799, Lmo2085, and Lmo0514. Quantitative PCR was performed using 2.5 μl of cDNA, 10 mM of each primer, and 12.5 μl of Platinum SYBR Green qPCR Supermix (Platinum SYBR Green qPCR Supermix–UDG with ROX, Invitrogen) in a final volume of 25 μl per reaction. The cycling parameters for quantitative PCR were as follows: 95°C for 30 s, followed by 40 cycles of 95°C for 5 s, 63°C for 20 s, and 72°C for 30 s. The primer sets used are listed in Table 1. Data was analyzed using LightCycler® 96 SW 1.1 software.

## 3. Results

### 3.1. Construction of rliB defective *L. monocytogenes*

**Fig 1:**
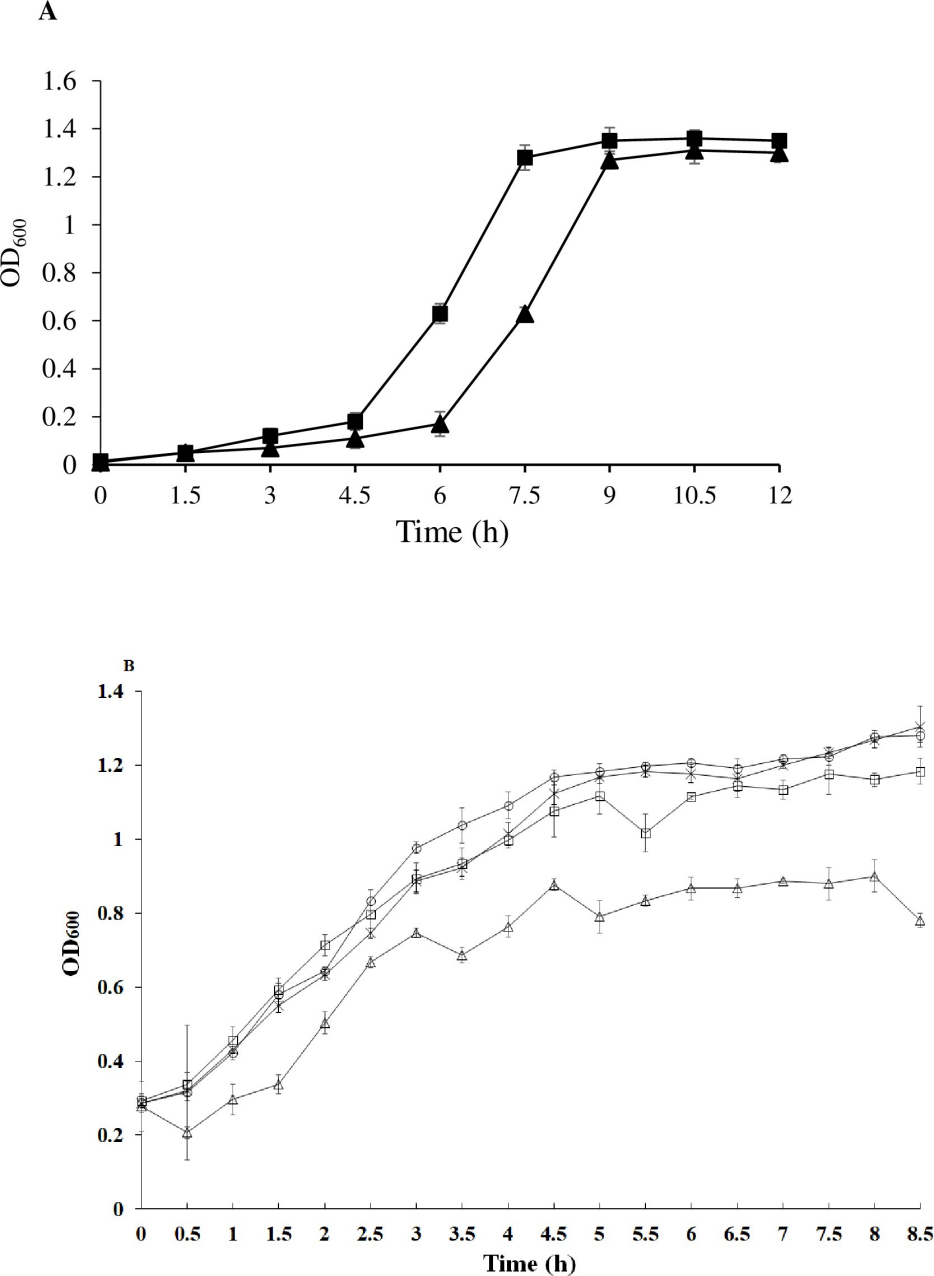
Growth curves and phage lysis effect in host strain. (A) Lm3-22-Δ*rliB* mutant (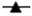) and wild-type Lm3-22 (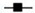) were inoculated on BHI agar (Cm, 10 μg/mL) and cultured overnight at 37°C. (B) Host bacteria were infected with phages at an MOI of 100, and the concentration (OD_600_ value) of host bacteria was determined every 30 minutes. Lm3-22 control (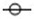), Lm3-22-Δ*rliB* control (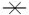), Lm3-22 with phage (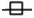), and Lm3-22-Δ*rliB* with phage (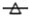). Values correspond to the mean from three independent experiments.

*RliB* defective mutants were constructed by homologous recombination and named Lm3-22-Δ*rliB*. The results showed that the *rliB* sequence was not detected in the mutant strain Lm3-22-Δ*rliB* after 14 successive subcultures, which indicated that the *rliB* sequence was stably deleted in Lm3-22-Δ*rliB* cells. It can be seen that the growth trend of Lm3-22-Δ*rliB* was consistent with that of the wild-type strain Lm3-22 at 37°C. The wild-type strain entered logarithmic phase after 4.5 h, while the Lm3-22-Δ*rliB* strain entered logarithmic phase after 6 h (Fig 1). The deletion strain was slightly slower than the wild strain, but there was no significant difference (*P* > 0.05).

### 3.2. Contribution of rliB sequence to the susceptibility of *L. monocytogenes* mutant to phage vB-LmoM-SH3-3

**Fig 2:**
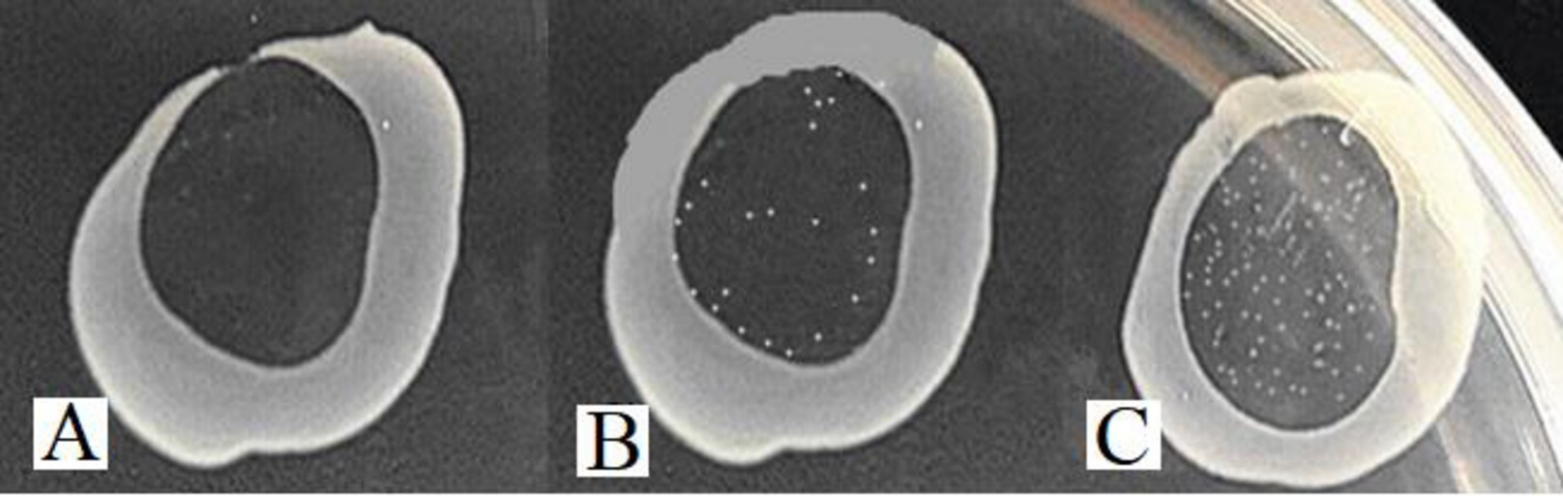
Characterization of *L*. *monocytogenes* phage sensitivity, drop assay using wild-type Lm3-22 and *rliB* deficient Lm3-22-Δ*rliB*. Phage vB-LmoM-SH3-3 was dropped on top of the deficient strain Lm3-22-Δ*rliB* (A), the wild-type strain Lm3-22 (B) and complemented strain Lm3-22-Δ*rliB*::B (C). Phage vB-LmoM-SH3-3 formed clear and regular transparent spots on the upper layer of Lm3-22-Δ*rliB*, while bacteria clone growth still existed in the inhibition circle of Lm3-22 and Lm3-22-Δ*rliB*::B, and the sensitivity of phage was stronger in Lm3-22-Δ*rliB* as compared with Lm3-22 and Lm3-22-Δ*rliB*::B.

**Fig 3:**
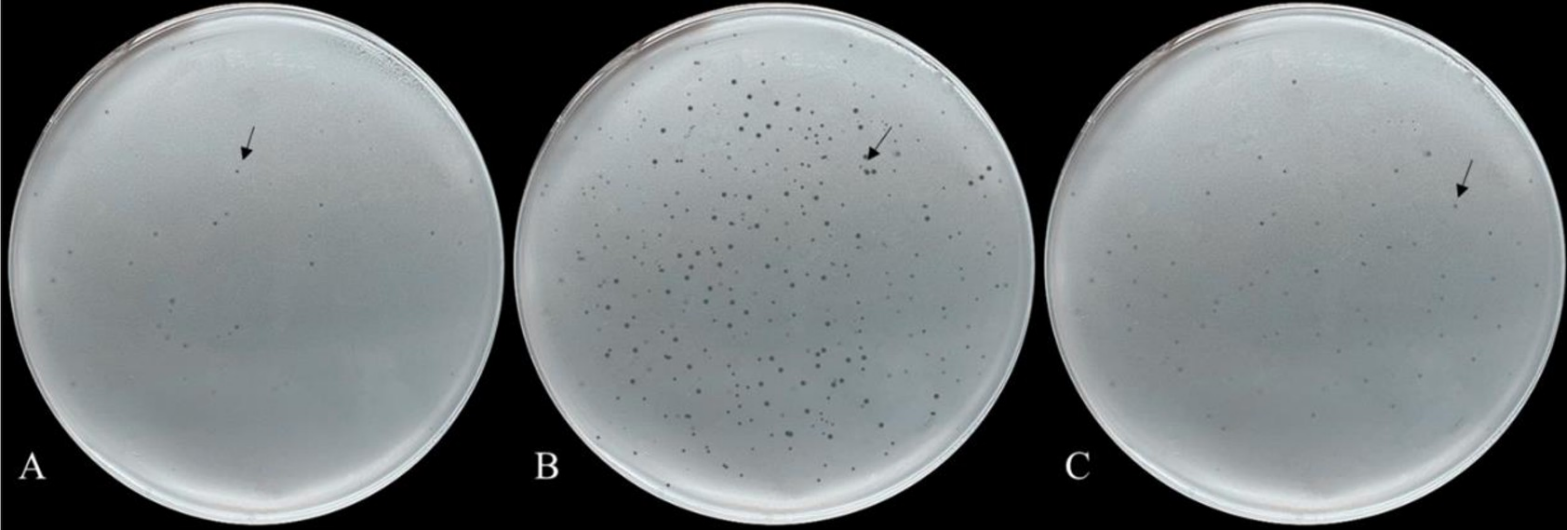
Efficiency of plaque formation. Phage lysate was mixed with Lm3-22, Lm3-22-Δ*rliB* and Lm3-22-Δ*rliB*::B. The resulting plaques were observed on double-layer plates. (A) Phage plaque formed using Lm3-22. (B) Phage plaque formed using Lm3-22-ΔrliB. (C) Phage plaque formed using Lm3-22-Δ*rliB*::B.

We further determined if there are any differences in the phage susceptibility between Lm3-22 and Lm3-22-Δ*rliB* strains. Our results showed that efficiency of plaque formation (EOP) of Lm3-22-Δ*rliB* versus the phage vB-LmoM-SH3-3 was 128 times higher than that of Lm3-22 (Table 2). There was no significant difference in EOP between complemented strains and the wild-type Lm3-22. These results suggesting that the *rliB* deficiency played a role in the interaction between phage and host strain. Moreover, after a phage drop was applied to the surface of a lawn of Lm3-22-Δ*rliB*, there were more clones in the inhibition zone of wild-type Lm3-22 after 24 h incubation compared with the mutant strain Lm3-22-Δ*rliB*. Also, clones in the inhibition zone of complementated strain Lm3-22-Δ*rliB*::B were are equal to Lm3-22 (Fig 2). We also found that mutant Lm3-22-Δ*rliB* was more effective and formed clearer plaques on double-layer plates, compared with wild-type Lm3-22 cells (Fig 3). No significant difference could be seen from complementated strain Lm3-22-Δ*rliB*::B compared with wild-type Lm3-22.

**Table 2:** EOP of Lm3-22-ΔRliB with phage vB-LmoM-SH3-3.

Strain	Titer (PFU/mL)	EOP
Lm008*	1.98×10^9^	1.0
Lm3-22	5.6×10^1^	2.8×10^−8^
Lm3-22-Δ*rliB*	7.2×10^3^	3.6×10^−6^
Lm3-22-Δ*rliB*::B	7.3×10^1^	3.69×10^−8^

EOP were detected by phage vB-LmoM-SH3-3. Phages were reacted with Lm008, Lm3-22-Δ*rliB*, Lm3-22 and Lm3-22-Δ*rliB*::B, respectively. The titers of phage vB-LmoM-SH3-3 from different host bacteria were determined, respectively. The EOPs were calculated by ratio of tested strain to reference strain and determining the average for three independent assays.

* Lm008 as reference strain.

### 3.3. Ad hesion, invasion, and intracellular proliferation

**Fig 4:**
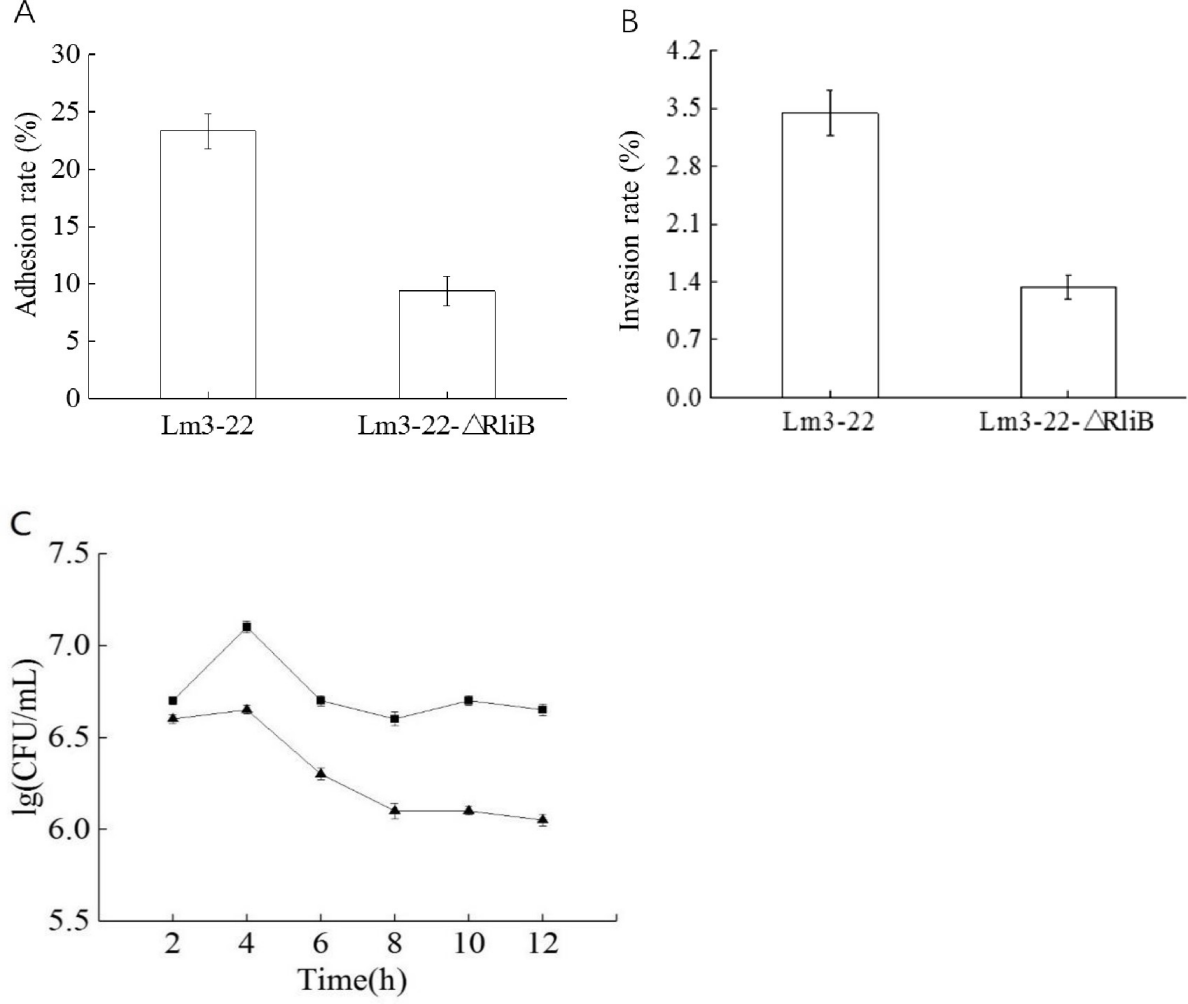
Cell adhesion, invasion, and intracellular proliferation ability of *rliB* deficient *L*. *monocytogenes*. The number of wild-type Lm3-22 (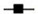) and *rliB* deficient Lm3-22-ΔrliB (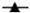) in RAW264.7 cells at various times after infection. Adhesion (A), invasion (B), and intracellular proliferation (C) were evaluated in the mouse macrophage cell line RAW264.7. The experiments were repeated three times, each time in triplicate wells for Lm3-22 and Lm3-22-ΔrliB, respectively. Values correspond to the mean from three independent experiments.

Cell adhesion, invasion, and intracellular proliferation were next examined in the macrophage cell line RAW264.7. The adhesion rates of Lm3-22 and Lm3-22-Δ*rliB* to the macrophage RAW264.7 cells were 23.33% and 9.37%, respectively. The adhesion ability of the defective mutant was significantly reduced. Similarly, the invasion ability of the mutant to RAW264.7 cells was also significantly decreased. Furthermore, the intracellular proliferation in RAW264.7 cells infected with Lm3-22 and Lm3-22-Δ*rliB* is shown in Fig 4(C), where the number of Lm3-22 and Lm3-22-Δ*rliB* both decreased within 4 h of infection. After 4 h, the number of bacteria decreased, and the level of decrease of Lm3-22-Δ*rliB* was more significant (P < 0.05), indicating that the deletion of *rliB* significantly affected the proliferation of Lm3-22 in cells.

### 3.4. Transcription levels of surface protein genes in rliB defective mutants infected with phage

**Fig 5:**
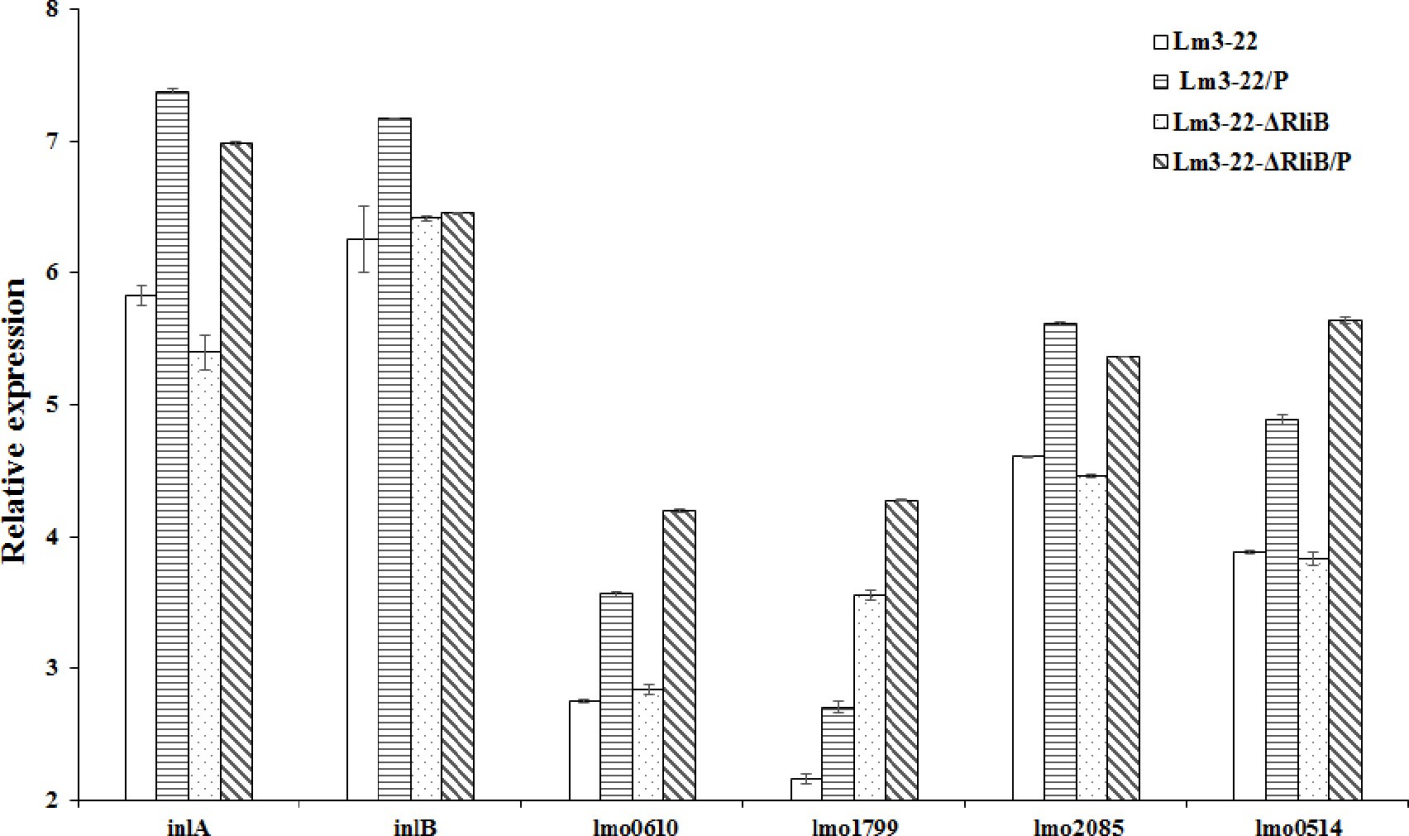
The relative expression levels of six surface protein coding genes in *L*. *monocytogenes*. Lm3-22 and Lm3-22-ΔrliB without phage were the control group. Experiment groups had phage added and were incubated at 37°C for 120 min. Data was analyzed using LightCycler® 96 SW 1.1 software. Mean fold-change values of triplicate independent experiments and standard deviation are shown.

Several listerial surface proteins including LPXTG motif, GW motif, and LRR protein, play key roles in listerial interactions with mammalian host cells. Therefore, we detected intracellular putative cell wall-associated genes *inlA*, lmo0610, lmo0514, lmo1799, lmo2085, *inlJ* (lmo2821), all of which encode products containing the LPXTG motif [2,3]. Internalin encoding gene *inlB* (lmo0434), which encode products containing the GW motif, was bind by way of non-covalent interactions mediated by their carboxy-terminal domains. The results of the qRT-PCR showed that the transcription level of the surface proteins coding gene *InlA*, *InlB*, Lmo0610, Lmo0514, Lmo1799, and Lmo2085 were all increased after interaction with phage in wild-type Lm3-22 (Fig 5). The expression of the genes *InlA*, *InlB* and lmo2085 in Lm3-22-Δ*rliB* were not as higher as in Lm3-22. However, the transcription levels of Lmo0610, Lmo0514, and Lmo1799 were significantly high in the Lm3-22-Δ*rliB* strain compared to the Lm3-22 strain (P < 0.05) (Fig 5). These results suggest that the *rliB* gene plays an important role in the interaction between phage and host strain.

## 4. Discussion

*L*. *monocytogenes* is ubiquitous in nature and can often be resistant to disinfectants. This fact contributes to the persistence of *Listeria* in food-associated environments, leading to the subsequent risk of food contamination and transmission to humans [27]. Moreover, a continuing increase of antibiotic resistance of *L*. *monocytogenes* has been reported [28] and antibiotic therapy fails in about >30% of cases. Phage as antimicrobials against pathogenic bacteria have become increasingly studied with regards to their application in many areas [29]. Notably, phages have been proposed as disinfectants to remove and prevent biofilm formation in clinical [30] and food-related environments [31,32], as well as in foods such as fruits, Ready-To-Eat (RTE) products, cheese, and milk [23,33–35].

In the present study, the characteristics of the *L*. *monocytogenes* isolate Lm3-22 (1/2a) from a slaughtering and processing environment was analyzed. It has low reaction activity with phage and weak plaque forming efficiency (Fig 3). Sequencing showed that *rliB* and the flanking sequences of Lm3-22 were 100% homologous to *L*. *monocytogenes* EGD-e. We analyzed the characteristics of the *rliB* deficient strain Lm3-22-Δ*rliB*, which was generated by homologous recombination. The *rliB* deletion of Lm3-22 could enhance the sensitivity to the phage vB-LmoM-SH3-3. The EOPs of the *rliB* defective strain Lm3-22-Δ*rliB* was 128 times higher than that of the -type strain Lm3-22, and plaques of Lm3-22-Δ*rliB* were clearer and more transparent on double-layer plates. Bacteria can avoid being infected by phages in several ways [36], such as adsorption blocking and impeding the entry of phage DNA. However, adsorption of mutant Lm3-22-Δ*rliB* was similar with wide-type Lm3-22. In this study, the enhanced sensitivity of phages to *rliB* deficient strains may have been related to the destruction of these mechanisms. Otherwise, bacteria that had already acquired resistance to antibiotics, persister, or biofilm cells could be killed by such an engineered phage and reduce the emergence of antibiotic-resistant mutants [37]. Therefore, we believe that *rliB* can regulate the interaction mechanism between phage and host.

Non-coding RNAs mostly function as coordinators of adaptation processes in response to environmental changes, the deletion of which may play an important role in regulation when they impact the interaction of phage and host bacteria. Bacterial ncRNAs are 50–400 nt long and located in intergenic regions. They play biological regulatory roles, mainly through base pairing and targeting of mRNA binding [38]. With increasing research, it has been found that non-coding RNA is an important regulator of bacterial metabolism, quorum sensing [39], biofilm formation, outer membrane protein formation, LPS modification, and regulation of virulence gene expression [40], all of which plays an important role in the regulation of bacterial gene expression in response to environmental changes, and is closely related to bacterial pathogenicity. Toledo Arana et al. found that *r1iB* mutants enhanced the colonization of *L*. *monocytogenes* in the mouse spleen [18]. In our study, we found that the invasion and adhesion of *rliB* defective strain were significantly decreased, and their proliferation ability in RAW264.7 cells was also significantly reduced. These probably were the important underlying mechanisms, as the absence of *rliB* reduced invasion capacity in *L*. *monocytogenes* virulence. Therefore, *rliB* is involved in *L*. *monocytogenes* virulence [18].

Virulence is primarily determined by many virulence genes (e.g., *inlA* and *inlB*). With these genes, *L*. *monocytogenes* is able to effectively adhere and invade host cells, proliferate, and spread between cells. InlA was also the first LPXTG protein to be identified, and contains a conserved LPXTG sequence motif [41]. Forty-one genes encoding LPXTG proteins have been found in the *L*. *monocytogenes* genome to date, which are mostly involved in virulence [42]. The transcription levels of virulence-related genes were determined here after Lm3-22-Δ*rliB* interaction with the phage vB-LmoM-SH3-3. Our results revealed that the absence of *rliB* could affect the transcription of virulence related genes *in vitro* while interacting with a phage. Lmo1799 encodes a peptidoglycan binding protein, with 226 Ala-Asp (AD) tandem repeats, which was significantly increased after *rliB* deletion. When phage infected this strain, the expression level showed an upward trend. It should be noted that the expression levels of lmo0514 (Internalin), lmo0610 (Internalin), and lmo1799 in Lm3-22-Δ*rliB* were significantly higher than those in Lm3-22 after phage infection. The AD of lmo1799 predicted to span the cell wall and expose the ligand-binding region at the bacterial surface [43], which may contribute to the binding of phages to host receptors and enhanced the phage sensitivity. InlB is similar to internalin, with a signal peptide and LRRs, but it does not possess an LPXTG motif [4], which reduced after *rliB* deletion wehther with or without phage treatment. Lmo0514 and lmo0610 are predicted to encode internalin-like protein, which encode products containing the LPXTG motif [44], were increased after phage treatment. Therefore, we suggested that the upregulation of LPXTG protein in the interaction between phage and host contributes to the sensitivity of phage. Thus, it is inferred that ncRNA *rliB* may indirectly regulate the interaction between phage and host by regulating the expression of LPXTG protein. However, the regulatory mechanism is still not clear. Attachment of phage particles to receptors on the bacterial surfaces is the initial step of phage infection and requires specific recognition of surface structures. It has been shown previously that adsorption of *Listeria* phages depends on the presence of specific sugar residues in the WTA [45–47]. Bioinformatic analysis of lmo1080, which is located upstream of the *rml* locus, revealed a putative N-terminal glycosyltransferase. Inactivation of *lmo1080* resulted in the loss of rhamnose, meaning that a phage cannot recognize the host strain [48]. There was no significant change in the expression of *lmo1080* in a *rliB* deficient strain, no matter whether it was attached to phage or not. This suggested that the enhanced phage sensitivity induced by *rliB* deletion is not directly related to the adsorption of phage. Moreover, Listeria ncRNA also acts directly on the phage, e.g, listeriophage ϕLS46 encode an anti-CRISPR protein that inactivated the type VI-A CRISPR system of Listeria seeligeri [49].

RliB plays an important role in the regulation of *Listera* virulence, however, its function in regulating phage and host interaction has not been reported. More than 300 types of ncRNAs have been found in *L*. *monocytogenes*, most of which can match the complementary noncontiguous regions of targeted mRNAs to regulate the expression of related genes. Therefore, in further studies, we plan to identify the phage target proteins or RNA of ncRNAs in the process of regulating the interaction between host and phage, so as to clarify the key role of ncRNAs in antimicrobial effects and therapy of phage in the future.
